# Vaccination Communication Strategies and Uptake in Africa: A Systematic Review

**DOI:** 10.3390/vaccines12121333

**Published:** 2024-11-27

**Authors:** Winifred Ekezie, Beauty Igein, Jomon Varughese, Ayesha Butt, Blessing Onyinye Ukoha-Kalu, Ifunanya Ikhile, Genevieve Bosah

**Affiliations:** 1 Centre for Health and Society, Aston University, Birmingham B4 7ET, UK; feyiigein@gmail.com (B.I.); joephilipvarughese@gmail.com (J.V.); 2 College of Life Sciences, University of Leicester, Leicester LE1 7RH, UK; 3 NIHR Biomedical Research Centre, Department of Respiratory Sciences, University of Leicester, Leicester LE1 7RH, UK; ayesha.butt45711@gmail.com; 4 School of Medicine, University of Nottingham, Nottingham NG7 2UH, UK; blessing.ukoha-kalu@nottingham.ac.uk (B.O.U.-K.); ifunanya.ikhile1@nottingham.ac.uk (I.I.); 5 Department of Media, University of Hertfordshire, Hatfield AL10 9AB, UK; g.bosah@herts.ac.uk

**Keywords:** Africa, vaccination, communication, information, views and practices, barriers and facilitators

## Abstract

**Background**: African countries experience high rates of infectious diseases that are mostly preventable by vaccination. Despite the risks of infections and other adverse outcomes, vaccination coverage in the African region remains significantly low. Poor vaccination knowledge is a contributory factor, and effective communication is crucial to bridging the vaccination uptake gap. This review summarises vaccination communication strategies adopted across African countries and associated changes in vaccine uptake. **Methods:** A systematic search was conducted in five bibliographic databases between 2000 and 2023 and supplemented with an additional Google Scholar search. Studies with data on vaccination communication and uptake in the English language were considered. A narrative synthesis was performed, and findings were presented in text and tables. **Findings:** Forty-one studies from fourteen African countries met the inclusion criteria. Several communication strategies were implemented for 13 different vaccines, mainly childhood vaccines. Mass campaigns and capacity building were the most common strategies for the public and health workers, respectively. Community-based strategies using social mobilisation effectively complemented other communication strategies.Overall, vaccination uptake increased in all countries following vaccination communication interventions. Barriers and facilitators to optimising vaccination communication at systemic and individual levels were also identified. Key barriers included lack of vaccine information, access issues, and high cost, while facilitators included improved vaccine education, reminders, trust-building initiatives, and community involvement. **Conclusions:** This review highlights effective vaccination communication strategies implemented across Africa as well as systemic and individual barriers and facilitators influencing vaccination uptake. The findings can inform strategies for vaccination communication and campaign planning to improve vaccination coverage in Africa.

## 1. Introduction

Africa has one of the highest rates of infectious disease burdens, with several emerging and re-emerging infections in the 21st century [1]. Between 2001 and 2022, more than 1500 public health emergencies, predominantly involving emerging infectious diseases, were reported, underscoring the critical need for effective and efficient prevention and management actions, such as vaccination [2]. Vaccination remians one of the most effective and cost-efficient public health interventions, preventing about 5 million deaths annually [3]. However, despite the advances in vaccine development, millions still do not access vaccination services in the African region. Hence, Africa lags behind in meeting the 2030 global immunisation targets, with approxomately 12 million children missing one or more vaccinations between 2019 and 2021 [4,5].

Vaccination efforts in Africa face by numerous systemic and individual challenges [6,7]. Systemic issues in supply and delivery include logistic difficulties such as maintaining the cold chain, transportation, and stock monitoring; inadequate coordination among stakeholders; and shortage of healthcare workers [8]. At the individual level, low uptake and missed vaccinations have been associated with geographical location, financial constraints, lack of awareness, trust, and vaccine hesitancy [8,9,10,11]. Vaccine hesitancy, in particular, delays or prevents vaccination uptake. One of the“5 Cs” model proposed to tackle hesitancy included the importance of communication in addressing vaccine hesitancy (Confidence, Complacency, Convenience, Communication, and Context) [12].

Studies indicate that effective communication is essential to bridging the vaccination uptake gap, increasing awareness, and combating hesitancy, with evidence showing that ineffective vaccination communication contributes to incomplete or zero-dose vaccination for African children [13]. Previous studies employing the 5Cs framework in Africa have often used aversion that excludes communication but focuses on slightly different parameters (Confidence, Complacency, Constraints, Calculation, and Collective responsibility) [14]. However, these studies have identified that reasons for low vaccination rates were often associated with gaps in communication strategies used for conveying information about vaccines [15,16,17]. Limited communication channels and resources can further hinder the efforts to counteract negative information about vaccines and build community trust [18]. Conversely targeted effective communication, which provides easy-to-understand vaccine information and strong evidence, increases uptake [19]. This, in turn, provides both individual and population protection from vaccine-preventable diseases (VPDs), which have high transmissibility, morbidity, and mortality rates. Creating effective public health messages is therefore critical for improving vaccine uptake in Africa.Several vaccination programs have been implemented acrossAfrican countries to address infectious diseases [20,21]. However, existsing reviews of communication strategies have predominantly focused on human papillomavirus (HPV) vaccines [7,22]. Given the disruptions caused by the COVID-19 pandemic, it is imperative to reassess vaccination communication strategies across Africa in order to enhance uptake for a wider range of VPDs. This systematic review aims to summarise commonly adopted vaccination communication strategies across African countries and evaluate their influence on vaccine uptake, with the goal of improving vaccination coverage and public health protection.

## 2. Methodology

The review protocol was pre-registered on the International Prospective Register of Systematic Reviews (PROSPERO ID: CRD42020196073) [23]. The review followed the Preferred Reporting Items for Systematic Reviews and Meta-Analyses (PRISMA) guidelines [24]. We conducted a comprehensive search of peer-reviewed literature in consultation with a medical librarian.

### 2.1. Eligibility Criteria

All population groups are at risk of vaccine-preventable infectious disease, so they were all considered in this review. The interventions of interest were vaccine communication strategies, acceptance, and uptake. The exposure of interest was the spread of vaccination education and information. Pre-defined eligibility criteria for inclusion included (i) research articles reporting vaccine communication and quantitative uptake evidence, (ii) studies conducted in African countries only, and (iii) studies published in English from 2020 to date. All study designs were included if they met the inclusion criteria. Exclusion criteria included systematic reviews, studies without quantitative outcomes related to vaccination communication (e.g., percentage of who engaged in the communication and vaccination uptake or coverage), and in vitro, non-human studies, non-English language publications, and studies not focused on African countries.

### 2.2. Search Strategy

We searched the following databases from 2000 to October 2023: MEDLINE, EMBASE, CINAHAL, Scopus, and Web of Science, along with a free text search of the first ten pages of Google Scholar. Only English-language publications focused on African countries were considered. The search terms were based on a combination of keywords for three key concepts: “Vaccination” AND “Communication” AND “African countries”. Within each concept, keywords were combined with Boolean search operators (see Supplementary S1), and the reference lists of eligible studies were also searched to identify additional relevant studies.

### 2.3. Study Selection

One reviewer carried out the literature search, and references were uploaded to the Rayyan review manager [25]. After the automatic removal of duplicates, the remaining studies were screened manually. All titles and abstracts were screened by two authors independently, and discrepancies were resolved through discussions with another author. References that met the inclusion criteria at the title and abstract screening stage then underwent full-text screening independently by two reviewers, and the reference lists of the included studies were also reviewed for additional relevant articles.

### 2.4. Study Appraisal

Quality assessment was conducted using the Joanna Briggs Institute (JBI) checklist that matched the design of the included studies with one reviewer conducting the assessment and a second reviewer verifying it [26]. The JBI quality assessment does not involve assigning numerical scores to checklist components, so the grading decision in this review was subjective. If a criterion did not apply to a specific study type, it was marked as ‘not applicable’ and did not contribute to the final quality score. Studies were graded based on how well they met the assessment requirements: weak (0–39%), moderate (40–79%), and strong (80–100%).

### 2.5. Data Extraction

Data from all included studies were extracted into Microsoft Excel. Key variables extracted included author(s), year, country, study population, design, vaccines administered, vaccination communication method, uptake, barriers, and facilitators. One reviewer performed the extraction, and another checked it for accuracy. Measures of effect extracted included proportions and confidence intervals, average means, standard deviations, prevalence ratios, and other applicable measures, as reported in the included studies.

### 2.6. Analysis and Narrative Synthesis

Findings from the included studies were entered into tables and descriptively synthesised following the SWiM guideline [27]. The primary outcome assessed was the vaccination communication approaches and their associated changes in vaccination uptake. The secondary outcomes explored were barriers, facilitators, and other factors influencing vaccination decisions, as well as changes in attitude after exposure to information provided through the identified vaccination communication methods. Comparisons across the different countries were also assessed. The analysis explored the variation in the outcomes of interest, but a meta-analysis investigation was not conducted due to high heterogeneity in the reported effects.

## 3. Results

### 3.1. Results of the Search

**Figure 1:**
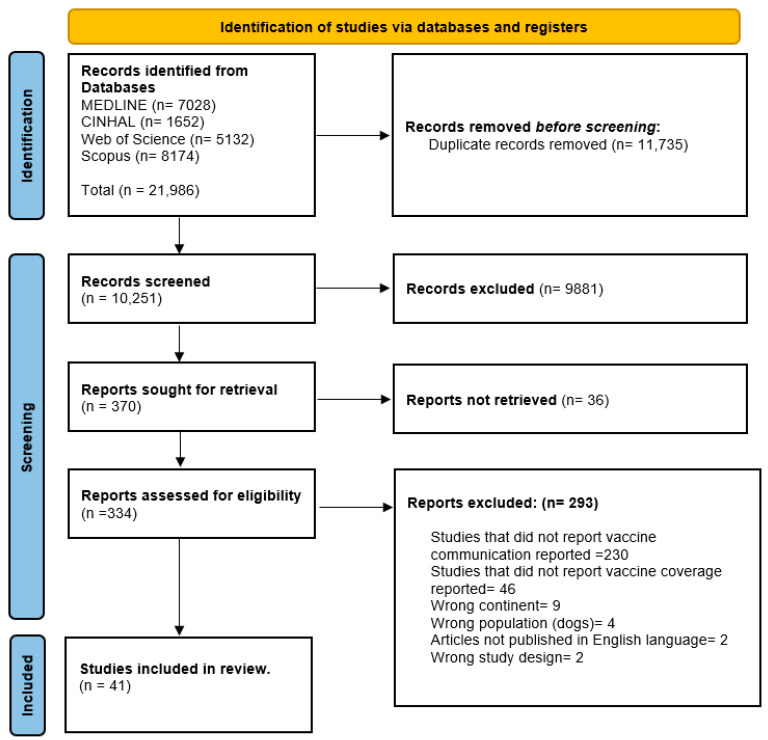
PRISMA flow diagram of study inclusion.

A total of 21,986 search results were identified from all data sources; 11,735 duplicates were removed, 10,251 titles and abstracts were screened, and 370 studies were identified for full text review(Figure 1). Finally, 41 studies were included in the review.

### 3.2. Study Characteristics

Table 1 presents a summary of the included publications. Fourteen African countries were represented: Nigeria (*n* = 15 studies), Ethiopia (*n* = 4), Ghana, Kenya, South Africa and Cameroon (*n* = 3 each), Malawi and Tanzania (*n* = 2 each), and one study from the Central Africa Republic, Egypt, Madagascar, Mali, Morocco, and Zimbabwe. The studies were conducted in three distinct settings: community (*n* = 23), health centres/clinics (*n* = 9), school (*n* = 7), and community and health centres (*n* = 2). A variety of study designs were adopted, including quasi-experiments (*n* = 18), cross-sectional (*n* = 6), randomised controlled trials (*n* = 9), and qualitative (*n* = 4) approaches. Additionally, there was also one article each of a documentary, program evaluation, government report, and commentary. The included studies were conducted between 2001 and 2023, with study durations ranging from 1 to 12 months (average = 5 months), with the longest durations found in the experimental studies [28,29,30,31]. The focus of the studies was mainly on vaccines for polio (*n* = 15 studies), human papillomavirus (HPV) (*n* = 9), Bacillus Calmette–Guérin (BCG) (*n* = 9), measles (*n* = 8), and COVID-19 (*n* = 6). Other vaccines included pentavalent vaccines (*n* = 5), diphtheria, tetanus, and pertussis (DPT) and Hepatitis B (*n* = 3 each); yellow fever, oral cholera, Pneumococcal conjugate vaccine (PCV) (*n* = 2 each), and one study each on tetanus toxoid and typhoid vaccine.

The reviewed studies encompassed a wide range of age groups, from infants under one year to individuals over 75 years old, with most studies targeting children and adolescents. Engagement with these groups was often through their caregivers, particularly mothers [32,33,34]; while a few studies also involved fathers and teachers [35,36]. Studies on vaccines for adults were primarily targeted at womenwith other studies focusing on adults in general [37], conflict victims [38], health workers, policymakers, and leaders [34,39].

Most studies were assessed as moderate quality (strong =14, moderate = 24, weak = 2). The potential risk of bias was primarily related to the intervention component details, outcome reporting (e.g., response rates, measures used), and statistical methods (e.g., accounting for confounding factors). Given the significant heterogeneity across the included studies, an in-depth statistical analysis of the findings could not be conducted. Therefore, the findings should be interpreted with caution.

**Table 1:** Summary of vaccination communications and uptake in the included studies.

Author	Country of Study	Study Design/ Study Duration	Vaccine Administered	Target Group/ Sample Size	Setting	Vaccination Communication Method	Vaccination Uptake	Quality Assessment
Abd Elaziz et al. 2010 [40]	Egypt	Quasi-experimental 2 months	Measles and rubella (MR) vaccine	Medical students (*n* = 341)	School	Campaign using posters	**Vaccine uptake:** 65%	Strong
Addi et al. 2021 [41]	Morocco	Cross-sectional (DNR)	COVID-19 vaccine	General population (*n* = NR)	Community	Official Ministry of Health website mass media	**Vaccine coverage:** 8.94%	Weak
Admassie et al. 2009 [42]	Ethiopia	Quasi-experimental 4 months	DPT 3-doses Polio 3-doses BCG Measles	Households (*n* = 3095)	Community	Training of health extension workers House visits	Fully vaccinated with BCG, polio, measles, and DPT vaccines is significantly larger in the treatment villages	Moderate
Akande et al. 2005 [43]	Ghana	Cross-sectional 1 month	Polio vaccine	Children aged 0–59 months (*n* = 3737)	Community	House visits Announcements by radio and gong-gong Mobile van Health worker’s education	**Vaccination uptake:** 98.8%	Moderate
Amani et al. 2023 [44]	Central African Republic	Cross-sectional 5 months	COVID-19 vaccine	General population (*n* = 5,570,659)	Community	Capacity building and training of CHW Health education by trained CHWs	**Vaccination coverage change:** 9% to 29%	Moderate
Amani et al. 2021 [45]	Cameroon	Cross-sectional (Evaluation) 1 month	Oral cholera vaccine (OCV)	General population (*n* = 537,274)	Health centre	House visits by social mobilisers Messages circulated through press, radio, sports, television, megaphones, banners, and posters	The overall vaccination coverage was 99.9%	Strong
Amare et al. 2021 [46]	Ethiopia	Quasi-experimental 7 months	Penta-3 Polio 3 vaccines	Health workers (*n* = 90)	Health centre	Capacity building of health workers	17.4% increase for Penta 3 vaccine in IG over CG	Moderate
Andrianarivelo et al. 2001 [47]	Madagascar	Quasi-experiment (DNR)	Oral polio vaccine (OPV)	Children up to 59 months (*n* = 929)	Community	Mass immunisation campaigns	**Vaccination coverage after two mass campaigns:** - 91.9% (113/123) children without a history of previous routine dose - 83.3% (20/24) children with a history of 1 or 2 routine dose(s) - 93.2% (150/161) children with 3 routine doses - 96.3% (158/164) children with 4 routine doses	Moderate
Appiah et al. 2022 [48]	Ethiopia	RCT 3 months	Penta 3 vaccine	Mothers of children (*n* = 638)	Community	**Radio Campaign:** **IG:** 10 + 10 + 30 (10 min radio drama on infant vaccination, 10 min discussion by CHW, 30 min phone-in from listeners) **CG:** No intervention	CG = 324; IG = 314 **Vaccine coverage change** **IG:** 89.8%, 95% CI: 85.9–92.9%) **CG:** (65.1%, 95% CI: 59.7–70.3%).	Strong
Ateudjieu et al. 2022 [37]	Cameroon	Cross-sectional 2 months	Oral cholera vaccine	General population (*n* = 9212)	Community	Training workshops for healthcare workers	First round = 4372, second round = 4840 **Vaccination coverage** - **First round:** 81.0% - **Second round:** 88.8% (single dose change 4.3%, second dose change 80.1%)	Strong
Bangure et al. 2015 [49]	Zimbabwe	RCT 7 months	Childhood immunisation	Women (*n*= 304)	Health centre	**IG:** Automated text reminders were sent at 6, 10, and 14 weeks **CG:** Routine health education	IG = 152, CG = 152 **Immunisation coverage** - **At 6 weeks:** IG (97%) vs. CG (82%), (*p* < 0.001). - **At 14 weeks:** IG (95%), CG (75%)	Moderate
Basheer et al. 2021 [50]	Nigeria	Quasi-experiment 4 months	Childhood immunisation	Mothers of children 12–23 months of age (*n* = 420)	Community	Training of primary HCWs to improve interpersonal communication skills	IG = 210, C = 210 **Routine childhood immunisation uptake (IG vs. CG)** - **Fully immunised:** 53.8% vs. 9.5% - **Partially immunised:** 16.6% vs. 32.8% - **Un-immunised:** 29.5% vs. 57.6% *p* < 0.001.	Moderate
Botha et al. 2015 [51]	South Africa	Quasi-experiment (DNR)	HPV vaccine	Girls (*n* = 2046)	School	Advocacy for school stakeholders and teachers Health education targeted at caregivers and schoolgirls Written information leaflets	- **At least one dose of HPV vaccine:** 99.2% - **Received 3 doses:** 87.8% - **Sufficient vaccination:** 91.6% of the vaccinated cohort	Moderate
Brown et al. 2016 [39]	Nigeria	RCT (DNR)	Childhood immunisation	Children aged 0–12 months paired with their mothers (*n* = 595)	Health centre	Cell phone call reminder Primary healthcare immunization Providers’ training intervention	**Immunisation completion:** - **IG (3 arms):** cell phone reminder (98.6%), PHC providers’ training intervention (70%), combined cell phone reminders and training of PHC providers (97.3%) - **CG:** 57.3%	Moderate
Crippin et al. 2022 [52]	Mali	Quasi-experiment 7 months	HPV vaccine	Women (*n* = 500)	Community and health centre	Community educational sessions by community health workers and a storytelling cloth	**Willingness of women to want their children to be vaccinated:** 87%	Moderate
Dougherty et al. 2020 [29]	Nigeria	Quasi-experiment 12 months	BCG Polio vaccine Hep B vaccine	Children aged 0–23 months and their mothers (*n* = 2639)	Community	Training of traditional barbers (direct and interpersonal communication)	**Received yellow card:** IG (16.6%)	Moderate
Dreyer et al. 2015 [32]	South Africa	Quasi-experiment (DNR)	HPV vaccine	Primary school girls; parents and female guardians (*n* = 3465)	School	Health education with presentation and leaflets	**Vaccination uptake** - **First dose:** 99.3% - **Second-doses:** 95.9% (974/1016) - **Third-dose:** 91.6% (1859/2030). - **Overall completion:** 90.5%	Moderate
Durrheim et al. 2001 [53]	South Africa	Commentary (DNR)	Polio vaccine Measles vaccine	Children (*n* = NR)	Community	Mass immunisation campaigns	- **Decrease in second-round national OPV coverage by:** 1995 (11.7%), 1996 (12.8%), 1997 (3.5%) - **Polio mass campaign coverage declined** by 7.7% between 1995 and 1997 (chi-square for trend = 7465.3, df =1, *p* < 0.001) - **Measles Campaign:** 1996 (91.1%) and the 2000 campaign (91.0%)	Weak
Egbon et al. 2022 [54]	Nigeria	Cross-sectional 5 months	HPV vaccine	School girls 9–19 years old (*n* = 100)	School	Community mobilisation and advocacy	**Vaccination completion:** under-14 years (60%, 42/70), ≥15 years 83% (25/30)	Strong
Ekhaguere et al. 2019 [28]	Nigeria	RCT 12 months	Penta 1, 2, 3 vaccine	Mothers of children (*n* = 600)	Health centre	Automate reminders using phone calls and text messages	IG (*n* = 300), CG (*n* = 300) **Completion of 12-month immunisation series:** IG (74%) vs. CG (66%); (RR: 1.12, 95%CI: 1.01–1.25; *p* = 0.03) **Received Penta-3 within 1 week of the expected date:** IG (84%) vs. CG (78%); (RR: 1.09, 95%CI: 1.01–1.17, *p* = 0.04) **Received measles immunisation within 1 week of the expected date:** IG (73%) vs. CG (65%); (RR: 1.33, 95%CI: 1.02–1.26, *p* = 0.02)	Strong
Gibson et al. 2017 [31]	Kenya	RCT 12 months	Pentavalent and Measles vaccine	Caregivers of infants aged 0–34 days (*n* = 2018)	Community	IG1 = SMS only (Financial incentives) IG2 = SMS + 75 KES IG3 = SMS + 200 KES	IG1 (*n* = 476), IG2 (*n* = 562), IG3 (*n* = 491), CG (*n* = 489) **- Full immunisation among children 12 months of age:** - **IG (3 arms): SMS only** (86%), **SMS + 75 KES** (86%), **SMS + 200 KES** (90%) - **CG:** 82% - **Coverage at 12 months and timely vaccination:** **- IG (3 arms): SMS only** (48%), **SMS + 75 KES** (60%), **SMS + 200 KES** (62%) - **CG:** 41%	Moderate
Jones and Kawesa-Newell, 2021 [55]	Malawi	Quasi-experiment (DNR)	HPV vaccine	School girls (*n* = NR)	School	Mini magazine	**Vaccine uptake change** - **Cohort 1:** 83% vs. 70%, *p* = 0.0028 - **Cohort 2:** 82% vs. 62%, *p* = 0.0002	Moderate
Kaduru et al. 2023 [33]	Nigeria	Quasi-experiment 5 months	Childhood immunisation	Caregivers (women) (*n* = 216)	Community	Community drama/theatre	**Fully immunised:** Baseline (46%), midline (55%), post-intervention (84%)	Strong
Levine et al. 2021 [56]	Ghana	RCT 5 months	Polio vaccine BCG vaccine	Mothers who delivered a live-born, surviving infant; primary caregivers (*n* = NR)	Community	IG1 = Voice call reminders IG2 = Community health volunteers and financial incentives (training of community health volunteers)	**Vaccination coverage** - **First dose of Polio and BCG:** higher in all arms than during the baseline period - **Vaccinated on time with both vaccines:** voice call reminder (37.8%), CHV and incentives arms (54.5%) - **Increase from pre-intervention to post-intervention:** voice call reminder (12.8% points), CHV and incentives arms (42.0% points)	Moderate
Meiring et al. 2019 [57]	Malawi	Quasi-experiment 3 months	Typhoid conjugate vaccine	Community members (*n* = NR)	School	Community mobilisation and advocacy Announcements and jingle for invitation for vaccination with a mobile van	School-based vaccine campaign increased community participation, exceeding recruitment targets (average, >200 children/day)	
Mekonnen et al. 2021 [30]	Ethiopia	RCT 12 months	Penta 3 Measles	Mothers and infant (*n* = 434)	Health centre	IG = Text message reminders CG = Routine verbal reminders	IG = 217, CG = 217 - **On-time vaccination:** IG (63.3%), CG (39.9%) - **Penta 2 coverage:** IG (98.1%), CG (95.3%) - **Penta 3 coverage:** IG (95.8%), CG 185 (86.9%) - **Measles vaccine coverage:** IG (91.5%), CG (79.3%)	Strong
Mohammed et al. 2023 [58]	Ghana	Cross-sectional (DNR)	COVID-19 vaccine	HCWs (*n* = 424)	Health centre	Media	**First dose of COVID-19 vaccine uptake:** 73.6%	Moderate
Mphuru et al. 2022 [59]	Tanzania	Qualitative 2 months	HPV vaccine	14-year-old girls (*n* = 10)	Community and Health centre	Health education in schools Community advocacy and sensitisation Mass media using posters, brochures, fliers, TV, radio, social media	- **First dose (HPV1) coverage:** 78% - **Second dose (HPV2) coverage:** 49%.	Strong
Msunyaro et al. 2023 [60]	Tanzania	Quasi-experiment 8 months	COVID-19 vaccine	Community members (*n* = 1,351,320)	Community	Community engagement; house visits, village meetings by community champions	**Increase during the campaign:** 10% to 22%	Moderate
Obi-Jeff et al. 2022 [34]	Nigeria	Qualitative (DNR)	Routine childhood immunisation (BCG, OPV, DPT, Hep B, Measles, Yellow fever vaccines)	Program stakeholders (policymakers, program managers, development partners) and HCW (*n* = 144)	Community	Automated text messages using immunisation reminders system	Many noted that personalised reminders reminded caregivers, especially those who were busy and forgot their child’s vaccination dates and prompted them to go for vaccination	Moderate
Odunyemi et al. 2018 [61]	Nigeria	Quasi-experiment (DNR)	HPV vaccine	Married civil servants’ women (*n* = 146)	Community	IG = Health education by nurses CG = No intervention	IG = 69, CG = 77 **Mothers were ready to accept HPV vaccination for their adolescent daughters** - **Baseline:** IG (73.9%), CG% (83.1%) - **3-months:** IG (93.8%), CG% (60.8%)	Strong
Oku et al. 2017 [62]	Nigeria	Qualitative 2 months	Childhood immunisation	Caregivers and Healthcare workers (*n* = 84)	Community	Health workers education Mass media (radio and jingles) Town announcers in church Home visits	All caregivers: -Expressed messages received in the clinic were useful -Received information about managing vaccination side effects	Moderate
Ozohu-Suleiman et al. 2010 [63]	Nigeria	Cross-sectional (DNR)	Polio vaccine	Community members, including caregivers (*n* = 2253)	Community	Mass immunisation campaigns using interpersonal sources	- **Immunisation campaign acceptance:** 5.1% - **Immunisation campaign resistance:** 44.9%	Moderate
Sato and Takasaki, 2021 [64]	Nigeria	RCT 2 months	Tetanus toxoid vaccine	Pregnant women/women with children (*n* = 1600)	Community	Graphical illustrations using flipcharts	IG = 782 **Decreased vaccine take-up** by 3.7–6.1%	Strong
Sato and Titus, 2021 [65]	Nigeria	Quasi-experiment 10 months	Childhood immunisation	Women with children (*n* = 515)	Community	IG = One-time tailored information on their children’s current vaccination status and the next schedule for vaccination CG = Generic information on the vaccination schedule	IG = 198, CG = 317 **Immunisation uptake**: IG (38.4%), CG (46.6%)	Strong
Ugwuoke et al. 2021 [38]	Nigeria	Quasi-experiment (DNR)	COVID-19 vaccine	Victims of conflicts in IDPS (*n* = 470)	Community	IG = Graphical illustrations using visual illustrations on the importance of COVID-19 vaccination and counselling CG = No intervention	IG = 235, CG = 235 **Intention toward COVID-19 vaccination (pre vs. post-test)** (mean, SD): IG (1.1, 0.21) vs. (3.8, 0.98), CG (1.2, 0.11 vs. 1.3, vs. 0.23)	Moderate
Vermandere et al. 2015 [35]	Kenya	Qualitative 2 months	HPV vaccine	Teachers and parents of girls (*n* = 7)	School	Training of teachers by HCWs Health education to create awareness for girls and fathers	Teachers = 4, Fathers = 3 Despite high baseline acceptance, reported uptake at follow up was low	Moderate
Wamai et al. 2012 [66]	Cameroon	Cross-sectional 2 months	HPV vaccine	Parents of girls aged between 9–13 years old (*n* = 337)	Community	Sensitisation campaigns conducted through local media using both radio and television	**Overall to vaccinate daughters:** 49.9% **Strong willingness to vaccinate:** 27.0%	Strong
Warigon et al. 2016 [67]	Nigeria	Quasi-experiment (DNR)	Polio vaccine	Children (*n* = 5991)	Community	Advocacy for religious leader support Local drama Media, radio, newspapers. and TV	**Received vaccination:** 85.5%	Moderate
Yau et al. 2023 [68]	Nigeria	Quasi-experiment (DNR)	Routine childhood immunisation (BCG, OPV, DPT, Hep B, Measles, Yellow fever) vaccines	Newborns (0–14 days old) and mothers (*n* = 435)	Health centre	IG = Color-coded bracelets CG = No bracelets, but they received standard information provided at routine immunisation services	IG = 256, CG = 179 **Completed their vaccination schedule at the fifth contact:** IG (62%), CG (41%), *p* < 0.0001	Moderate
Yego et al. 2023 [36]	Kenya	RCT (DNR)	COVID-19 vaccine	High-risk patients either due to age (>60 years old) and/or have a documented medical history of hypertension or diabetes (*n* = 8514)	Health centre	IG1 = Phone call + Gain messages IG2 = Phone call + Loss messages IG3 = Phone call + Social norms messages IG4 = SMS + Gain messages IG5 = SMS + Loss messages IG6 = SMS + Social norms messages CG = No intervention	IG1 = 1216, IG2 = 1216, IG3 = 1216, IG4 = 1216, IG5 = 1216, IG6 = 1217 CG = 1217 1716 (47%) participants in the phone call channel received intervention **Vaccination status of those who received the intervention:** 83% (1524) - **Completed vaccine doses:** 89% (1267) - **Partially vaccinated:** 11% (157) had been	Strong

**NOTE:** BCG = Bacillus Calmette–Guérin, CG = control group, CHW = community healthcare workers, DNR = duration not reported, DPT = diphtheria, tetanus, and pertussis, HCWs = healthcare workers, Hep B = hepatitis B, HPV = human papillomavirus, IG = intervention group, OCV = oral cholera vaccine, RCT = randomized controlled trial RR = relative risk, VPD = vaccine-preventable disease.

### 3.3. Study Findings

#### 3.3.1. Vaccination Communication Methods

Most studies used multiple vaccination communication methods, especially mass vaccination campaigns [47,53,63]. The verbal communication mediums included radios [43,45,48,59,62,66,67], television broadcasts [45,59,66,67], and social media [59]. Direct verbal communication was also utilised through home visits, social mobilisers, community mobilisation, announcements, and advocacy [43,44,51,54,57,59,62,67]. Seven studies used information from print media, such as newspapers [67], posters [40,45,47,59], flyers and leaflets [51], and magazines [55]. Two studies used graphical illustrations as a communication method for vaccination [38,64]. Automated reminders to get vaccinations were also used through text messages [28,30,31,34,36,49], phone calls [28,39,56], or a combination of both [28,36].

Health education was the key element of most vaccination communication [32,49,51,52,59,61]. This also included capacity building and training of healthcare and vaccination providers to improve interpersonal communication [35,37,39,42,43,44,45,46,50,56] and education for other groups, including other community members, such as traditional barbers and caregivers [29,35,51].

Community engagement approaches used included advocacy with stakeholders [51,54,57,59,67], house visits [42,43,44,45,60,62], and social mobilisation [44,45]. Other methods used to spread vaccination information included using community drama to share vaccination messages through entertainment [33,67] and using a five-colour-coded bracelet representing different immunisation contacts given to newborns [68]. Open-door announcements around the communities were an integral part of disseminating vaccination-related information [43,57].

#### 3.3.2. Vaccination Communication Sources and Purposes

The information included in the vaccination communication was from various contributors. However, the most common sources were the national Ministries of Health [30,37,40,41,44,45,50,54,59]. Non-governmental organisations (NGOs), foundations, and trusts were also significant information sources [45,52,54,55,57]. On a broader scale, journalists and media outlets were essential in shaping public perception [45,67]. The role of the media was underscored by studies [38,47,58,62,63], research teams [28,46] and wider sub-national public health and surveillance sectors also played a role in vaccination communication [31,45,51,54,57]. Training manuals and recommendations from organisations like the World Health Organization (WHO), the Centers for Disease Control and Prevention (CDC), etc., were critical resources for developing communication strategies and resources [31,35,39,59,68].

The purpose of most vaccination communication was to inform and educate the public about either the disease targeted by the vaccine and/or the vaccine itself [29,32,33,41,43,45,48,51,52,54,56,57,58,60,61,62,64,66,67]. Another commonly reported communication aim was to ensure timely vaccination compliance using the reminder system [28,30,31,39,40,49]. Developing and enhancing vaccine providers’ skills were recognised as a crucial reason for implementing vaccination communication training [37,44,46]. Some vaccination communication efforts were implemented to improve vaccine uptake and acceptance [38,45,50,55,59], including encouraging caregivers to vaccinate their children [29,36]. In one study, three distinct messaging strategies—gain messaging, loss messaging, and social proofing—were used via phone calls or text messages to promote vaccination among high-risk populations [36].

### 3.4. Vaccination Coverage and Uptake

Overall, vaccination coverage and uptake increased after the implementation of the vaccination communication interventions in all countries. In Nigeria, most studies showed a clear increase in vaccination coverage [28,29,33,34,39,50,54,63,68]. A cross-sectional study on HPV community mobilisation and advocacy showed that 60% (42/70) of girls under 14 and 83% (25/30) aged 15 and older completed their vaccinations [44]. Another study aimed at newborn identification and referral by traditional barbers reported a 16.6% increase in the number of individuals in the intervention group who received a yellow card at the end of the study [29]. Conversely, Sato and Takasaki reported a decline in vaccine uptake by up to 6.1% despite increased participants’ perceived risk of disease after viewing graphical illustrations of the tetanus toxoid vaccine [64]. Some other studies also showed no significant change in vaccine coverage rates after the communication intervention [38,65].

All four studies in Ethiopia showed increased vaccination coverage [30,42,46,48]. The two intervention studies for Penta-3 vaccines showed a significant increase in vaccination coverage [46,48]. The uptake after a radio campaign was 89.8% compared with 65.1% in the non-intervention group and 17% higher in the Penta-3 vaccine after health worker capacity building in the intervention group compared with the non-control group [48]. Another study reported a significantly higher proportion of children getting fully vaccinated with BCG, polio, measles, and DPT after trained health extension workers paid home visits [42].

The three studies in Kenya also reported significant vaccine uptake changes [31,35,36]. Gibson et al. reported that 86% of children successfully followed up for pentavalent and measles vaccines were fully vaccinated [31]. Similarly, another study reported an 83% increase in COVID-19 vaccine coverage [36]. In contrast, another study reported low vaccine uptake at follow-up [35].

All studies conducted in Ghana showed an increase in vaccine coverage [43,56,58]. For example, Mohammed et al. reported a 73.6% uptake of the COVID-19 vaccine’s first dose after a media intervention in health centres [58]. Two studies in Cameroon indicated a high vaccination coverage against cholera. Amani et al. reported an overall vaccination coverage of 99.9% after a mass vaccination campaign against cholera [45]. Another study in Cameroon observed a significant 8% rise in oral cholera vaccination coverage, with rates increasing from 81.0% in the first round of the vaccination campaign to 88.8% in the second round, following the training of vaccination providers during the program’s monitoring and evaluation process [37]. However, a study by Wamai et al. showed a low willingness to be vaccinated against HPV in Cameroon among parents of girls aged between 9 and 13 years old (49.0%) [66].

The study in Zimbabwe showed an increase in vaccine uptake among children of mothers who received SMS reminders compared with children of mothers who did not [49]. The immunisation coverage at six weeks was 97% in the intervention group and 82% in the non-intervention group (*p* < 0.001) [49]. Similarly, the study in the Central Africa Republic showed that COVID-19 vaccine coverage tripled from 9% to 29% with the capacity-building of community health workers and other communication efforts [44]. The single study for Mali showed that the willingness of women to vaccinate against HPV increased to 93% after a community education campaign [52]. Also, a study in Egypt reported a moderate vaccination uptake of 64.8% after a poster campaign targeted medical students against measles and rubella [40].

The study in Madagascar reported that overall seroprevalences for all poliovirus types increased significantly among the children after the mass campaign: 78.2% vs. 94.3% for poliovirus type 1, 86.0% vs. 98.5% for poliovirus type 2, and 69.1% vs. 89.8% for poliovirus type 3 [47]. The Tanzania study showed coverage of the first dose of HPV vaccine (HPV1) at the end of 2019 was 78%, and the second dose of HPV vaccine (HPV2) coverage was 49% [59]. A rapid COVID-19 vaccine coverage reported a 12% increase (from 10% to 22%) in the number of people vaccinated after the community engagement campaign [60]. In contrast, the reported COVID-19 vaccine coverage in Morocco was relatively lower at 8.94%, even after an official mass media campaign by the Ministry of Health website [41].

Two studies in South Africa showed an increase in HPV vaccination [32,51]. One study reported an overall completion rate of 90.5%, with 99.3% of consented girls receiving the first dose of the vaccine [32]. Among those vaccinated, a higher proportion received the two-dose regimen (95.9%) than the three-dose regimen (91.6%) [32]. Likewise, Botha et al. showed that 91.6% of the girls in the vaccinated cohort achieved adequate vaccination coverage [51]. This is in contrast to the other studies by Ogunbanjo et al. in South Africa that reported a decrease in vaccination coverage for the polio and measles vaccine [53].

Findings from the two studies in Malawi were in a school setting. One study reported that girls exposed to the HPV vaccination communication had a higher vaccine uptake after reading Zathu mini magazine and were significantly more likely to have had their allocated doses of the HPV vaccine than those who had not (83% vs. 70%) [55]. The school-based vaccine campaign increased community uptake, exceeding recruitment targets to date (on average, >200 children/day) [57].

### 3.5. Barriers and Facilitators to Vaccine Uptake

Various barriers and facilitators to vaccination communication intervention and general vaccine uptake were identified in the included studies (see Table 2). In relation to the communication methods used, Bangure et al. stated that the overall increase in vaccine uptake was likely attributed to the use of SMS reminders [49]. Hence, a population with no access to mobile phones with the required features needed to deliver the intervention will not be able to benefit effectively from such intervention, as indicated in another study that stated lack of mobile phones and inability to read text messages were barriers to intervention participation [34]. These factors highlight the importance of compatibility and operability in vaccination communication delivery.

General barriers to vaccination compliance included a combination of facility and population factors. For instance, Ekhaguere et al. reported barriers to childhood immunisation uptake, including long waiting times, distance to a clinic, transportation costs, inconducive clinic sites, and mother’s forgetfulness [37]. The clinic environment, long waiting times, and health worker attitudes were also reported in other studies as obstacles to receiving vaccination information [62]. The health workers’ attitudes were affected by poor communication skills, lack of motivation, and community, including vaccine resistance attitudes [63]. Such vaccine hesitancy was reported as a significant barrier to uptake [41,69]. The primary barriers identified in the first round of vaccines with multiple doses were often related to low awareness and the absence of children [37,56]. Concerns about vaccine safety were particularly affecting COVID-19 vaccine acceptance and uptake [58,60]. Fear of vaccine effectiveness, lack of trust in vaccines, potential side effects, vaccine safety, lack of information regarding vaccine and venue and cost were barriers to HPV vaccine uptake [35,61,66]. One study in Nigeria showed low literacy as a barrier to vaccination uptake among community members [29], and another study reported controversial religious theories about vaccines that affected polio vaccine uptake [59,63]. Similarly, several studies reported a lack of appropriate information and misinformation as a significant barrier affecting vaccine uptake [37,40,48,59,61,67].

Facilitators to vaccine uptake included increased vaccine knowledge using acceptable information [34,40,66], free vaccination [41,58], health staff task shifting for improving equitable vaccine access [44], vaccination reminders, e.g., using text messages [31] and providing incentives for both vaccine administrators and community members [56]. Increased knowledge of vaccine was observed to be both a facilitator and barrier, as shown in two studies that reported awareness of the protective effect of HPV increased willingness for vaccination, but also information of possible dangers generated fear that the vaccine harms the fertility of girls [34,66]

Quantitative evidence of the positive and negative factors that influence vaccination uptake, even among health professionals, were observed in a study in Ghana, which found that factors motivating respondents to receive the COVID-19 vaccine included its lack of cost (82.7%), perceived efficacy, and safety (75.6%), protection against the virus (70.8%), the sense of social responsibility (61.9%), benefits outweighing the risks (72.1%), absence of harm (62.2%), and observing others getting vaccinated (62.2%) [58].

**Table 2:** Summary of vaccination facilitators, barriers, accessibility, availability, and service gaps.

Author, Year	Facilitators	Barriers
Abd Elaziz et al. 2010 [40]	More health education	Insufficient information, fear of getting infection, past infection, unconcerned, afraid of injections, vaccinated in private, fear of side effects, others
Addi et al. 2021 [41]	Vaccination campaign is free for all citizens and foreigners	Fake news in social media, low level of education and economic status, and insufficiency in population awareness programs
Amani et al. 2023 [44]	Task shifting provided equitable vaccine access	
Andrianarivelo et al. 2001 [47]	OPV doses during the mass vaccination campaign improved their immune status	
Appiah et al. 2022 [48]		Lack of communication and knowledge about the importance of vaccinations
Ateudjieu et al. 2022 [37]		Absenteeism (49.4%), Lack of awareness (18.2%)
Bangure et al. 2015 [49]	Overall increase likely attributed to use of SMS reminders	
Crippin et al. 2022 [52]		Seeking consent from husband or partner first
Dougherty et al. 2020 [29]	Parents trusted traditional barbers; messages shared consistently throughout the community reinforced their beliefs that the advice benefited child health	Low levels of literacy among community leaders and barbers
Egbon et al. 2022 [54]		Remote area, hard-to-reach locations, high operational costs, infrastructure health workforce, vaccine delivery included operational costs exacerbated by a lack of adequate health workforce and infrastructure
Ekhaguere et al. 2019 [28]		Long wait times (55%), transportation cost (34%), distance to the clinic (4%), forgetfulness (2.2%), clinic not conducive for mother and child (2%)
Gibson et al. 2017 [31]	SMS reminders plus monetary incentives were modestly effective	
Jones and Kawesa-Newell, 2021 [55]	Girls interest in reading the Zathu mini magazine as they were already familiar with the characters and believed the Zathu brand to be trustworthy and reliable	
Levine et al. 2021 [56]	- Vaccination provided by a community-level health system infrastructure - Incentives to both CHV and caregivers	Timely vaccination and complete birth registration are not remunerated and formally cadred
Mohammed et al. 2023 [58]	COVID-19 vaccine, vaccine-free (82.7%), sufficient efficacy and safety (75.6%), protects against the virus (70.8%), social responsibility (61.9%), benefits outweigh the dangers (72.1%), does not harm people (62.2%), seeing others taking it (62.2%)	Vaccine rapid development and approval (41.0%), immediate side effects (39.2%), unforeseen future effects (37.5%), health workers refusing to take the COVID-19 vaccine (24.5%)
Mphuru et al. 2022 [59]		Misinformation, religious radio station airing HPV vaccine causes infertility
Msunyaro et al. 2023 [60]		Vaccine safety concerns Frequent changes in COVID-19 vaccine types confusing
Obi-Jeff et al. 2022 [34]	- Perceived importance in reminding and educating community members on the importance of immunisation - Satisfaction with the message content, especially the health facility day and operation time accuracy	Lack of mobile phones and the inability to read text messages as barriers to intervention participation
Odunyemi et al. 2018 [61]		Lack of information deterred vaccinating daughters (85.5%), under age for vaccination, unaware of the venue of vaccination, cost of vaccine
Oku et al. 2017 [62]		**- Barriers affecting public:** clinic environment, long waiting times, health worker attitudes **- Barriers affecting health workers:** poor communication skills, poor motivation, and attitudes of community members, including vaccine resistance
Ozohu-Suleiman et al. 2010 [63]		Fears of polio vaccine contamination with anti-fertility hormone and HIV virus aimed at depopulating Muslims around the world
Vermandere et al. 2015 [35]	Protecting girls fertility	Bad experiences or rumours about other vaccines (polio and asthma especially) indicating possible danger of vaccines; new vaccine was potentially hidden experiment; fear vaccine harm girls fertility; fear vaccination will enhance sexual activity among children
Wamai et al. 2012 [66]	Knowledge of HPV	Concerns about vaccine effectiveness (31.8%) and side effects/safety (18.4%)
Warigon et al. 2016 [67]		Negative campaigns against polio vaccine in the media; negative campaigns initiated by scholars and religious leaders through various media platforms persuading audiences who were largely nonliterate vaccines were contaminated
Yego et al. 2023 [36]		No communication access via phone calls due to incorrect number and change in SIM

### 3.6. Vaccine Accessibility and Availability

Availability and accessibility of vaccination communication and services were not reported in most studies. The most common systemic reasons for vaccination attempts failing were vaccines not being available or stocked out or health providers refusing to open vials out of fear of wastage [56]. Also, hesitancy was higher when vaccination centres were too far and thus not easily accessible [37,47]. In contrast, where vaccines were readily available, e.g., for HPV, in addition to mass campaigns, increased potential for maximum vaccine coverage, as highlighted in Nigeria and Cameroon [37,61].

## 4. Discussion

To the best of our knowledge, this is the first study to present a systematic review of the communication strategies implemented to improve the uptake of a broad spectrum of vaccinations in Africa. The included studies represented 14 African countries, with several communications for children and adolescent vaccinations, particularly polio, HPV, and BCG. The identified adult vaccines were predominantly for mothers and COVID-19. Communication targeted at health workers aimed to improve their interpersonal communication skills with patients. Mass vaccination campaigns were the most common communication method, and other direct (e.g., home visits and text reminders) and indirect (e.g., training of community leaders who educated their communities) approaches were used. Community engagement was an essential part of indirect communication approaches. National health departments were the primary sources of the information; also, a few NGO interventions were identified, and both sources were guided by international vaccination guidelines. Vaccination coverage and uptake overall increased after exposure to the vaccination communication interventions in all the countries. However, factors like trust, vaccine availability, and accessibility significantly affected uptake. Barriers to uptake included lack of vaccine knowledge, vaccination services, distance to health centres, and vaccine costs at an individual and health system level. These factors contributed to high levels of vaccine hesitancy. In contrast, factors that improved uptake included increased vaccine knowledge, free vaccine services, and vaccination reminders.

The identified predominant vaccine focus aligned with a recent review study that highlighted the resurgence of VPDs, particularly polio, despite efforts to control them [70]. The significant barriers to vaccine uptake were a lack of awareness and information about vaccinations and concerns about potential side effects. Lack of vaccination knowledge is a crucial factor influencing vaccination in Africa [6], but this is also a significant issue among similar populations in other regions such as Europe [71]. Thus, this review points out the importance and potential of vaccine communication. However, the limited number of countries represented highlights the gap in African vaccination communication evidence. Similar limited country representation has also been observed in other review studies investigating vaccination in Africa [6,7]. Our review showed that the critical source of vaccination information communication is the national health departments; hence, countries need to invest in national-scale vaccination communication plans to improve population awareness, uptake, and sustained vaccine delivery, especially routine vaccinations for children and mothers, as well as new vaccines developed to tackle emerging and re-emerging diseases such as COVID-19. This finding supports other evidence that has shown populations expect national institutions to be responsible for fact-checking and verifying vaccination information [72]. Having adequate knowledge improves precautionary and vaccine uptake behaviour; hence, targeted and clear messaging on the protection that vaccines afford to personal health could motivate uptake and practices [73].

Although mass campaigns were the most common communication strategy, there was no clear evidence of the most effective type of campaign. Social media platforms were another medium of interest, but only limited studies showed this. Interpersonal relationships and word-of-mouth from both health workers and community leaders were also significant determinants of vaccination uptake, similar to another review in Africa [6,7,74]. The observed community mobilisation approaches were effective in reaching and engaging diverse communities due to the influence these groups of people have on the community members; thus, communication support and training are required for such nonexpert messengers [74]. Our findings also showed that in all countries, most people were willing to get vaccinated as a result of all forms of communication, and people were open to reminders and nudges to facilitate vaccination uptake. Overall, vaccination acceptance and uptake are likely to be enhanced through multichannel communication [19,75]. Thus, implementing well-designed vaccination communication strategies and interventions, such as the creative and innovative measures identified, e.g., the use of automated reminders, drama, town announcers, etc., could help increase overall vaccination coverage. For instance, Indigenous communication media such as town criers can be used in rural African communities [76], radio drama increases vaccination awareness and knowledge [77], while new media such as text messages, electronic reminders, and immunisation campaign websites have been extensively noted to increase immunisation coverage rates [77,78,79,80]. Adapting such nudge-based interventions is necessary to appropriately adapt how information is framed and delivered to intended audiences [81].

Childhood immunisation and some maternal vaccinations were the common vaccine focus, showing gaps in communication for adult vaccines. The lack of communication for adult vaccines could influence their perceptions of the importance of vaccines for children, especially adolescent girls [6,82]. Interactive communications with parents through workshops and presentations led by medical professionals and health workers were effective in boosting vaccination, similar to findings from other studies [6,83]. Also, evidence has shown a positive association between interactive social mobilisation and first-dose coverage [83]. Thus, supporting existing communication around routine and new vaccine recommendations would improve vaccine uptake [83,84].

Targeting all stakeholders involved in vaccination delivery, communication, decision-making, and acceptance is essential for achieving maximum vaccine uptake and completion rates. As recommended in the ‘Ten actions towards vaccination for all’, healthcare professionals and the media also need to be empowered to provide effective, transparent, and objective information to the public and fight false and misleading information [85]. Hence, a communication strategy incorporating appropriate and available communication tools should be part of every immunisation program [7]. One essential tool is healthcare workers, who are considered the most trusted expert messengers [74]. Thus, providing communication training to improve their interpersonal communication skills with patients is critical to help them become more assertive while practising shared decision-making and deliberation with their patients [22,86]. Other communication tools, including mobile phones, are used for message reminders. However, devices need to be available, compatible, and operable by users to avoid disengagement [34]. To ensure broader engagement, it is necessary to deliver information through various sources, target different groups, and provide tailored messages from the outset to address concerns, especially about experimental vaccines and their effects on other issues, such as fertility, to pre-emptively counter rumours and misinformation that negatively influence vaccination programs [72]. Such targeted, effective communication addresses most factors influencing vaccine hesitancy [74,87]. Previous studies have shown that public health communication has different engagement levels, which all carry potential risks, benefits, and challenges when embracing social components [88].

Improving communication will not be impactful without adequate vaccine availability. Beyond awareness and increased knowledge through vaccination communication, shortages in vaccine supplies and accessibility of vaccination sites significantly contribute to low vaccine coverage. Similar to other reviews, access to and experiences of vaccination services, including undesirable features of vaccination services, long waiting times at healthcare centres, and cost implications, influenced vaccination decisions and practices in Africa [6,89]. The review by Mavundza et al. articulates suggested questions to consider when planning and implementing interventions to reduce vaccine hesitancy and increase vaccine acceptance and uptake in Africa [6].

### 4.1. Implication of Findings

The findings from this review indicate that various communication strategies have been implemented across Africa and that appropriate vaccine communication activities can significantly improve vaccine coverage. However, there is a gap in vaccine communication evidence across the entire region, with the limited number of countries represented. Thus, all countries need to be more proactive in the development, implementation, and reporting of vaccine communication strategies. To overcome barriers like vaccine hesitancy, such communication needs to clearly outline the benefits and risks of vaccines so as to tackle concerns that encourage hesitancy. Embedding communication plans in immunisation programs, such as activities identified in this review for both the public and health workers, will boost acceptance and uptake. Using multiple communication channels and combinations, especially through community networks, will enhance the impact across multiple audiences. Vaccine uptake also depends upon national guidelines, healthcare workers, and supplies, and these factors need to be considered before any vaccination communication intervention is launched. Thus, addressing logistical challenges at a national level, such as the availability of vaccines and competent staff, distance to facilities, and vaccine cost, would help reduce the broader barriers to vaccination. Our findings can, therefore, help inform strategies that national vaccination campaigns may consider to improve vaccinations in Africa.

### 4.2. Strengths and Limitations

A key strength of this review is that the search was conducted to assess a wide range of vaccine-preventable diseases among various disadvantaged groups in all African countries to provide a broader assessment beyond the different countries. Considering that only 14 countries were represented, this may limit the generalisability of the findings across the continent. Our findings were also in line with other publications while highlighting significant gaps in the current body of evidence in Africa. The review was limited to peer-reviewed English-language publications only; hence, we may have missed some other relevant literature. Therefore, the data may be subject to publication bias. Grey literature that may have had relevant and interesting information was also excluded. Wide variations in study aims, intervention approaches, and countries reported made performing extensive comparisons and in-depth statistical analyses challenging. Several included studies employed multiple communication methods and sources, but most did not directly measure their individual impact on vaccination uptake and coverage, making it challenging to draw definitive conclusions about their effectiveness. More qualitative and quantitative research is required to investigate vaccine communication across different groups and countries in Africa to understand current communication strategies further and develop tailored vaccine communication intervention strategies for different populations and vaccines. Additionally, more innovative and improved vaccination communication services need to be developed for populations in Africa and beyond.

## 5. Conclusions

Our study underscores the significant impact of communication strategies on vaccine uptake in African countries. Findings from this review highlight the effectiveness of various approaches in increasing vaccination coverage across diverse populations. Systemic barriers, including inadequate vaccine knowledge, challenges with logistics, and vaccine hesitancy, were identified as major obstacles to effective immunisation. However, facilitators such as education, reminders, and accessible vaccination services showed substantial potential in improving vaccine acceptance and compliance. Findings from this systematic review provide a roadmap for public health campaigns to improve vaccine coverage and support Africa’s progress toward global immunisation targets.

## Supplementary Materials

The following supporting information can be downloaded at: https://www.mdpi.com/article/10.3390/vaccines12121333/s1, S1: OVID Medline review search.
