# Supplementary material for: Vaccination Communication Strategies and Uptake in Africa: A Systematic Review
Source: Vaccines (Basel). 2024 Nov 27;12(12):1333. doi: 10.3390/vaccines12121333 (PMC11679460; doi:10.3390/vaccines12121333)
Supplement: Supplementary file 1 [file vaccines-12-01333-s001.zip › vaccines-3268310-supplementary.pdf]

Vaccination Communication Strategies and Uptake in Africa: A Systematic Review

Supplementary S1: OVID Medline review search

1	exp Vaccines/
2	exp Vaccination/
3	exp Vaccinations/
4	exp Immunization/
5	exp Immunisation/
6	1 or 2 or 3 or 4 or 5
7	communicat*.mp.
8	campaign*.mp.
9	educat*.mp.
10	inform*.mp.
11	aware*.mp.
12	7 or 8 or 9 or 10 or 11
13	exp Algeria/
14	exp Angola/
15	exp Benin/
16	exp Botswana/
17	exp Burkina Faso/
18	exp Burundi/
19	exp Cameroon/
20	exp Cabo Verde/
21	exp Central African Republic/
22	exp Chad/
23	exp Comoros/
24	exp Congo/
25	Democratic Republic of Congo.mp.

26 exp Ivory Coast/

27 exp Djibouti/

28 exp Equatorial Guinea/

29 exp Egypt/

30 exp Eritrea/

31 exp Ethiopia/

32 exp Gabon/

33 exp Gambia/

34 exp Ghana/

35 exp Guinea/

36 exp Guinea-Bissau/

37 exp Kenya/

38 exp Lesotho/

39 exp Liberia/

40 exp Libya/

41 exp Madagascar/

42 exp Malawi/

43 exp Mali/

44 exp Mauritania/

45 exp Mauritius/

46 exp Morocco/

47 exp Mozambique/

48 exp Namibia/

49 exp Niger/

50 exp Nigeria/

51 exp Rwanda/

52 Saharawi Arab Democratic Republic.mp.

53 exp Sao Tome/ and Principe.mp. [mp=ti, ab, hw, tn, ot, dm, mf, dv, kf, fx, dq, bt, nm, ox, px, rx, ui, sy, ux, mx]

54 exp Senegal/

55 exp Seychelles/

56 exp Sierra Leone/

57 exp Somalia/

58 exp South Africa/

59 exp South Sudan/

60 exp Sudan/

61 exp Swaziland/

62 exp Tanzania/

63 exp Togo/

64 exp Tunisia/

65 exp Uganda/

66 exp Zambia/

67 exp Zimbabwe/

68 13 or 14 or 15 or 16 or 17 or 18 or 19 or 20 or 21 or 22 or 23 or 24 or 25 or 26 or 27 or 28 or 29 or 30 or 31 or 32 or 33 or 34 or 35 or 36 or 37 or 38 or 39 or 40 or 41 or 42 or 43 or 44 or 45 or 46 or 47 or 48 or 49 or 50 or 51 or 52 or 53 or 54 or 55 or 56 or 57 or 58 or 59 or 60 or 61 or 62 or 63 or 64 or 65 or 66 or 67

69 6 and 12 and 68

70 limit 69 to english language

71 limit 70 to humans

72 limit 71 to yr="2000 -Current"
